# A Covariance Matrix Reconstruction Approach for Single Snapshot Direction of Arrival Estimation

**DOI:** 10.3390/s22083096

**Published:** 2022-04-18

**Authors:** Murdifi Muhammad, Minghui Li, Qammer Abbasi, Cindy Goh, Muhammad Ali Imran

**Affiliations:** 1 James Watt School of Engineering, University of Glasgow, Glasgow G12 8QQ, UK; david.li@glasgow.ac.uk (M.L.); qammer.abbasi@glasgow.ac.uk (Q.A.); cindy.goh@glasgow.ac.uk (C.G.); muhammad.imran@glasgow.ac.uk (M.A.I.); 2 Research and Development Department, RFNet Technologies Pte Ltd., Singapore 319319, Singapore

**Keywords:** antenna array, direction-of-arrival, DOA, single snapshot, uniform linear array, ULA

## Abstract

Achieving accurate single snapshot direction of arrival (DOA) information significantly improves communication performance. This paper investigates an accurate and high-resolution DOA estimation technique by enabling single snapshot data collection and enhancing DOA estimation results compared to multiple snapshot methods. This is carried out by manipulating the incoming signal covariance matrix while suppressing undesired additive white Gaussian noise (AWGN) by actively updating and estimating the antenna array manifold vector. We demonstrated the estimation performance in simulation that our proposed technique supersedes the estimation performance of existing state-of-the-art techniques in various signal-to-noise ratio (SNR) scenarios and single snapshot sampling environments. Our proposed covariance-based single snapshot (CbSS) technique yields the lowest root-mean-squared error (RMSE) against the true DOA compared to root-MUSIC and the partial relaxation (PR) approach for multiple snapshots and a single signal source environment. In addition, our proposed technique presents the lowest DOA estimation performance degradation in a multiple uncorrelated and coherent signal source environment by up to 25.5% with nearly negligible bias. Lastly, our proposed CbSS technique presents the best DOA estimation results for a single snapshot and single-source scenario with an RMSE of 0.05° against the true DOA compared to root-MUSIC and the PR approach with nearly negligible bias as well. A potential application for CbSS would be in a scenario where accurate DOA estimation with a small antenna array form factor is a limitation, such as in the intelligent transportation system industry and wireless communication.

## 1. Introduction

Direction of arrival (DOA) estimation techniques as part of array signal processing have been intensively studied over the past few decades for a variety of applications such as wireless communications [1], radar [2], and vehicular systems [3,4]. A large number of high-resolution methods have been proposed [5,6,7,8,9,10,11,12,13]. The classical subspace-based methods such as the multiple signal classification (MUSIC) [14] and their variants [10,15,16] exploit the covariance matrix of the array output to determine the DOA estimation, which is known to be among the best type of DOA estimators. However, these techniques tend to be computationally expensive and are difficult to implement in real-world scenarios where cost is a concern [17,18]. In addition, there have also been attempts at implementing machine-learning (ML) algorithms to DOA estimation techniques as a state-of-the-art system [11]. In addition, many array input data snapshots are required to construct a sufficiently data-rich covariance matrix. This leads to a long overall computational time as more data snapshots are collected for accurate covariance matrix formulation and DOA estimation. Also, it is known that these techniques are severely degraded when signals are coherent. Thus, developing a single snapshot-based DOA estimator is an ongoing research challenge that must be fulfilled for fast and accurate DOA estimation.

Most of the proposed techniques suitable for real-time implementation have aimed at reducing the computational load of subspace decomposition per update but not the number of array snapshots necessary to attain a certain level of estimation performance [18,19]. Using a single snapshot has been challenging, as one of the major drawbacks is that the DOA estimator’s performance gets degraded for a reduced number of snapshots. Single snapshot DOA estimation techniques for efficient DOA computation have been researched in the literature but only perform well when the signal-to-noise ratio (SNR) is high [15,20,21]. The computational complexities have also been an active talking point when developing new DOA estimation methods. One of the simplest methods of reducing the computational load is the usage of a single instead of multiple snapshots to formulate the covariance matrix for DOA estimation.

Nevertheless, there have been multiple attempts at using a single snapshot that has provided comparable results compared to an estimator that utilizes multiple snapshots. In [20], a novel approach for recursively estimating the DOA using a single snapshot was proposed. A deterministic identification algorithm was used as a performance criterion which renders the DOA estimator robust against modeling error and additive noise via trial and error. A key drawback to this technique is that it is computationally expensive due to the algorithm’s high snapshot sampling requirements and recursive nature. In addition, the physical structural information of the array is required as part of the initialization parameters. It is highly susceptible to estimation errors when multiple signal sources of interest need a significantly large antenna array aperture. In [22], a low complexity single snapshot DOA estimation algorithm was proposed. It is first found that the conventional low-resolution discrete Fourier transform (DFT) spectrum effectively provides an initial estimation performance. Then, the proposed algorithm narrows down the search region for the angle of interest. However, this technique is effective in a massive uniformed linear array (ULA) geometry where the number of array elements is more than 128 to achieve the desired results. Therefore, the critical drawback of past proposed DOA estimation techniques is either an antenna geometry limitation that performs well at high SNR or is highly complex computationally. This limits the application in reality, with implementations only in high-budget cost applications such as military or large-scale aerospace industries [2,17].

Recently, there have been innovative methods that utilize ML with DOA estimation—colloquially dubbed as an ML-based class of DOA estimation. Reference [23] presents a good feasibility study of the recent progress and work of ML-based DOA estimators in automotive applications. Several deep learning models were compared and investigated for their suitability for automotive angle estimation, such as the deep convolutional neural network (CNN) and deep multi-layer perceptron (MLP) [11]. The models were trained with model and data-based approaches for data generation, including simulated scenarios and real-world measurements from 400 automotive radar sensors. These ML techniques were compared against several baseline angle estimation algorithms such as Bartlett, root-MUSIC, and the deterministic maximum likelihood (DML) DOA algorithms. Based on the study in [23], their analysis proved the viability of ML-based super-resolution DOA estimation for automotive radar with a single snapshot sample. However, it does come with significant drawbacks and limitations [23,24]. Large datasets for training these ML models for DOA estimation are required for all possible real-world scenarios. Training based only on synthetic data will result in poor estimation performance when testing with actual sensor data [25]. Second, the computational complexity of implementing ML-based DOA estimators is still highly complex, with the limited availability of public RF datasets for ML training [23]. Furthermore, even though ML training with datasets can be done offline, real-world scenarios are still unpredictable with multiple factors, such as weather, noise, and varying operating frequency [24,25]. Therefore, the ML model will require additional training to update and adapt to the new environment, which will impact operational efficiency.

Alternatively, the emerging field of sparse representation DOA estimation has aroused enormous attention. Based on the observation that signals impinging on an array are intrinsically sparse in the spatial domain, the DOA estimation problem can be formulated as a basis selection problem, where the basis entries are the discretized manifolds of a sensor array according to the angle of interest. In [26], a novel DOA estimator was proposed based on a novel data model using the concept of a sparse representation of array covariance vectors. DOA estimation was achieved by jointly finding the sparsest coefficients of the array covariance vectors on an overcomplete basis. Although simulation experiments have validated the high resolution and the capability to estimate coherent signals, the technique proposed in [24] is computationally complex and requires many snapshot samples to achieve good results. In [27], a novel Sparse Iterative Covariance-based Estimation (SPICE) DOA estimator was proposed to combat the need for multiple snapshots to reduce the computational complexity. The proposed approach was obtained by minimizing a covariance matrix fitting criterion and is particularly useful in multiple and single snapshot cases. Although their experiments show promising results, a key drawback is the need for a complex minimization solver to achieve reasonable estimates. In addition, a specific geometry of the antenna array is required to achieve the best DOA estimates. This results in a computationally complex DOA estimation technique and would require expensive hardware for real-world implementation.

Furthermore, as the covariance matrix plays an integral part in DOA estimation, the geometric-based class of estimators has been an active research field to improve DOA estimation accuracy further. The geometric class of DOA estimators is based on information geometry (IG), which constitutes a framework that measures the parameters’ closeness between different possible DOAs via the Fisher Information Matrix (FIM). The usage of IG for DOA estimation was proposed in [28]. The proposed method in [28] uses geodesic distances in the statistical manifold of probability distributions parametrized by their covariance matrix to estimate the DOA of several sources. Simulation results have shown that the proposed method provides an equivalent performance at high SNR against MUSIC and MVDR, lacking DOA estimation robustness and improved resolution capabilities at low SNR. In [29], an IG-based method called the string transform based information geometry (STRING) technique was proposed for DOA estimation and considered the relationship between the optimum scalar with unknown signal DOAs and powers and its linear relationship to tackle the problems faced in [28,29]. Based on the simulation results in [29], the STRING method achieved the best DOA estimation performance compared to MUSIC and MVDR. One drawback is that the best DOA estimation performance occurs when two sources are spaced close to each other. In addition, although it presents high DOA estimation accuracy, it lacks statistical bias and, therefore, lacks the predictability of its estimates.

This paper aims at developing a computationally efficient and accurate 1-D DOA estimation algorithm with a ULA antenna array geometry by exploiting the steering vector feedback and covariance matrix structure into the estimation and assuming the relationship between the number of sensors M and signal source, L is L<M. The fundamental characteristic of our proposed technique enables DOA estimation in many applications with cost, size, and hardware limitations, such as but not limited to the field of transportation and vehicular signal localization and high-bandwidth connectivity, especially in the current uprising of wireless communication [30]. We propose a simple approach consisting of a pre-processing covariance matrix reconstruction to determine a comparative steering vector by manipulating the structural information of the covariance matrix to improve DOA estimation performance. The computational efficiency is achieved using a single snapshot instead of multiple snapshots in our proposed algorithm to reduce data collection time while improving DOA estimation accuracy in a full range of SNR environments. Efficiency is achieved by using a predetermined DOA estimation stage using a root-MUSIC-like algorithm [8]. The derived DOA from the first stage is then used to determine the DOA initial estimates. This value is then used as feedback to determine the new steering vector. Finally, the final DOA estimation is then computationally retrieved via the reformulated covariance matrix. With a focus on lightweight design philosophy, our proposed method presents key features that compact single snapshot and high DOA estimation accuracy with low computational complexity in a wide SNR range. To that end, the critical advantage of our proposed method presents efficient covariance matrix data collection with a single snapshot coupled with good DOA estimation performance.

We provide extensive experiments to show the superiority of our proposed method compared with root-MUSIC and the state-of-the-art eigenvalue-based partial relaxation approach [13] in adverse scenarios. The scenarios demonstrated in this paper are in a low SNR environment ranging from 0 dB to 5 dB across all simulations. The simulation section presents the stable performance across a wide range of SNR environments, multiple signal source DOA estimation, and an exhausted single snapshot sample situation to showcase our proposed DOA algorithm performing higher estimation accuracy and statistical bias deviations. According to our simulation study in a static environment, it has been shown that our proposed technique supersedes the root-MUSIC and PR approach by up to 80.6% in multiple snapshots with a single signal source, 180% in multiple uncorrelated and coherent signal sources with multiple snapshots, all in terms of RMSE DOA estimation reduction. Furthermore, our proposed technique presents a 92.6% DOA estimation performance gain for single snapshot scenarios. We also provide the computational time and compare how this affects the DOA estimation application in a real-world setting.

The remainder of this paper is organized as follows. Section 2 presents the system model for an antenna array and the derivation of the crucial antenna array’s signal covariance matrix. Section 3 offers our proposed DOA estimation technique called the covariance-based single snapshot (CbSS) DOA estimator. Section 4 presents the simulation results and discussion demonstrating the performance of our proposed method in multiple and single signal sources and snapshot data under varying SNR. In addition, the effect of the parameters chosen is demonstrated. Finally, Section 5 concludes the paper.

## 2. Data Model and Problem Formulation

Consider a uniformed linear array (ULA) with M isotropic sensor elements receiving the incoming signals emitted by L narrowband far-field sources with unknown and distinct DOAs $\left\{\begin{array}{ccc} \theta_{1}, & \ldots , & \theta_{L} \end{array}\right\}$. Assuming that the number of signal sources, L is known and, L<M, the kth observation vector of the received signal is expressed as [17]:

(1) x(k)=As(k)+n(k) ,  k=1,…,K,

where $A=\left[a\left(\theta_{1}\right),a\left(\theta_{2}\right),\cdots ,a\left(\theta_{L}\right)\right]$ is the steering matrix of size M×L, $s\left(k\right)={\left[\begin{array}{ccc} s_{1}\left(k\right) & \ldots & s_{L}\left(k\right) \end{array}\right]}^{T}$ is the source signal vector with ${\left(\cdot \right)}^{T}$ being the transpose, K is the total number of snapshots, and $a\left(\theta_{L}\right)$ is the steering vector of the *L*th signal source, which can be expressed as

(2) $$a\left(\theta_{L}\right)={\left[\begin{array}{cccc} 1 & e^{j\frac{2\pi }{\lambda }\sin d_{2}\left(\theta_{L}\right)} & \ldots & e^{j\frac{2\pi }{\lambda }\sin d_{M}\left(\theta_{L}\right)} \end{array}\right]}^{T}$$

where λ=c/f is the wavelength of the carrier frequency, $d_{M}$ is the inter-element spacing distance being no greater than half the carrier frequency’s wavelength, λ/2, f is the signal carrier frequency, and c is the speed of light.

It is assumed that the noise vector n(k) is a spatially and temporally white Gaussian process with zero mean and covariance $\sigma_{n}^{2}I_{M}$ where $\sigma_{n}^{2}$ is the power and $I_{M}$ is the M×M identity matrix. Moreover, for this data model, it is assumed that the noise is uncorrelated with the signal sources, s(k). The main objective of our study is to estimate the L DOAs from the observations ${\left\{x\left(k\right)\right\}}_{k=1}^{K}$. Therefore, the theoretical covariance matrix of x(k), in matrix form, is given as [10]

(3) $$\begin{array}{ll} R_{xx} & =E\left\{X\left(k\right)X^{H}\left(k\right)\right\}\\ & =E\left\{\left(As+n\right)\left(s^{H}A^{H}+n^{H}\right)\right\}\\ & =AE\left\{s\cdot s^{H}\right\}A^{H}+E\left\{n\cdot n^{H}\right\}\\ & =AE_{S}A^{H}+E_{N}\\ & =AE_{S}A^{H}+\sigma_{n}^{2}I_{M}, \end{array}\;$$

where $E\left\{\cdot \right\}\;\mathrm{and}\;{\left(\cdot \right)}^{H}$ represent the mathematical expectation expression and the Hermitian transpose, $E_{N}=E\left\{n\cdot n^{H}\right\}$ and $E_{S}=E\left\{s\cdot s^{H}\right\}$ are the M×M noise and L×L signal source matrix subspaces, respectively.

However, the theoretical covariance matrix is unavailable in a real-world application, and an estimation is required. If we do not know the exact statistics for the signals and noise independently, we can assume that the process is ergodic. Therefore, $R_{xx}$ is replaced by the sample covariance matrix ${\hat{R}}_{xx}$, which is defined as [10]

(4) $$R_{xx}\approx {\hat{R}}_{xx}=\frac{1}{K}\overset{K}{\underset{k=1}{\sum }}x\left(k\right)x^{H}\left(k\right)=\frac{1}{K}XX^{H}$$

The key issues we face are the overall computational load time and the DOA estimation accuracy, which are yet to be addressed clearly, particularly in varying SNR environments [31]. The problem faced in a low SNR environment is challenging to distinguish the different subspaces and signal information. Our proposal in this paper addresses this issue, especially when faced with an array size limitation without sacrificing DOA estimation accuracy. In addition, we will also address the single snapshot limitations of DOA estimation by introducing our robust, high-resolution DOA estimator called the CbSS technique. The CbSS estimator is an all-encompassing DOA estimation algorithm robust in performance across a wide range of SNR with good functionality in estimation performance and computational time.

## 3. Covariance-Based Single Snapshot DOA Estimator

This section introduces the covariance-based single snapshot (CbSS) DOA estimator. We first provide a detailed theoretical estimation model based on the theoretical covariance matrix to identify the root cause of estimation error. Then, we determine the lower and upper bound of an optimum diagonal-loading factor value for error minimization. Lastly, based on the theoretical model estimation, we show how to minimize the error and noise suppression for practical single snapshot DOA estimation implementation.

### 3.1. Defining the Error Terms in Covariance Matrices

First, we want to highlight an apparent disparity in data information between the theoretical covariance matrix in (3) and the sample covariance matrix in (4) that eventually leads to DOA estimation performance degradation. As the number of snapshot samples is limited, the sample covariance matrix in (4) has inherent errors. Thus, (3) and (4) have a simple additive error mathematical relationship that can be written as

(5) $$\begin{array}{c} {\hat{R}}_{xx}=R_{xx}+\mu D \end{array}$$

where $R_{xx}$ is the theoretical covariance matrix in (3), D, is a zero-mean random matrix with unit variance, and μ is a constant that indicates the estimation error of the estimated covariance matrix.

In (5), the term μD represents the additive inherent error by the sample covariance matrix. The errors are the numerical differences between the theoretical and sample covariance matrices. Evidently, the larger the estimation error, the worst the DOA estimation performance will be as ${\hat{R}}_{xx}$ is numerically further away from the theoretical covariance matrix, $R_{xx}$. Clearly, μD is the leading cause of estimation performance degradation in the sample covariance matrix relative to the theoretical covariance matrix.

In past research studies, the diagonal loading method is a simple and efficient method for improving the robustness of an estimator that conducts matrix decomposition [32]. Thus, we introduce a data-dependent approach to determine an optimal diagonal loading factor. From (5), we can combine the sample covariance matrix with a diagonal loading value and the estimation error. Therefore, we include the diagonal loading parameter onto the sample covariance matrix in (5), which is defined as

(6) $$R_{DL}=R_{xx}+\mu D+\varepsilon_{DL}I$$

where $\varepsilon_{DL}$ is the additive diagonal loading factor of interest to improve the DOA estimation accuracy.

### 3.2. Determining the Lower & Upper Bounds of the Diagonal-Loading Factor for Error Minimization

Assuming that, at sufficiently high SNR values or a high number of snapshot samples, the theoretical covariance matrix and diagonal loading term combined are much larger than the inherent error, $∥R_{xx}+\varepsilon_{DL}I∥\;\gg \;\mu ∥D∥$, then we can exploit the orthogonal properties of (3) and (6) and identify the cause of error by taking the inverse of the diagonally loaded covariance matrix in (6). Taking into account the inverse matrix approximation properties, the inverse of (6) can be expressed as

(7) $$\begin{array}{ll} R_{DL}{}^{-1} & ={\left(R_{xx}+\varepsilon_{DL}I\right)}^{-1}{\left[I+\mu D{\left(R_{xx}+\varepsilon_{DL}I\right)}^{-1}\right]}^{-1}\approx {\left(R_{xx}+\varepsilon_{DL}I\right)}^{-1}\left[I+\mu D{\left(R_{xx}+\varepsilon_{DL}I\right)}^{-1}\right]\\ & ={\left(R_{xx}+\varepsilon_{DL}I\right)}^{-1}\left\{I-\frac{\mu }{\varepsilon_{DL}+\sigma_{n}^{2}}D\left[I-A{\left[A^{H}A+\left(R_{xx}+\varepsilon_{DL}I\right)E_{s}{}^{-1}\right]}^{-1}A^{H}\right]\right\}. \end{array}$$

The sample covariance matrix and theoretical covariance matrix are equal in a perfect scenario. However, due to the existing error terms in real-world scenarios where the sample covariance matrix is used, it is impractical to achieve zero error. Thus, the diagonal-loading factor is introduced to minimize error and noise terms. However, it is crucial to determine the lower and upper boundary values to not statistically skew the covariance matrix estimation, which is directly linked to the estimation of the DOAs of interest. If the diagonal loading factor lies beyond the boundary, it will result in poor estimation results, which is undesirable.

Therefore, based on the hypothesis, from (7), the terms inside the first brackets should ideally be a close non-zero value to the theoretical covariance matrix, which can be given as $R_{xx}+\varepsilon_{DL}I≅R_{xx}$. If $\varepsilon_{DL}I$ is set to zero, then no diagonal loading factor is used, particularly inside the curly brackets, and would result in the exact error-prone covariance matrix estimation. Due to the existing $\varepsilon_{DL}I$ and other error terms in the curly brackets, only a close value would be achievable for either a sufficiently high SNR or snapshots samples. Therefore, the diagonal loading value should be much smaller than the diagonal element value of the theoretical covariance matrix. This ideal assumption and a diagonal loading factor upper bound can be expressed as

(8) $$\varepsilon_{DL}\ll R_{xx}\left(i,i\right),\;\varepsilon_{DL}\neq 0.$$

where i represents values from 1 to M.

Next, we want to determine the lower bound in deciding the optimal diagonal loading factor. It can be observed that the leading cause of performance degradation by the second term is in the curly brackets in (7). Optimal performance is achieved if the second term equates to zero, which would minimize the estimation error in an ideal scenario. Therefore, to achieve minimal error, it is ideal to have the following hypothetical constraint,

(9) $$\frac{\mu }{\varepsilon_{DL}+\sigma_{n}^{2}}\ll 1.$$

Then, we rearrange the parameters of (9), which should then result in the following inequality,

(10) $$\varepsilon_{DL}+\sigma_{n}^{2}\gg \mu ,$$

where (10) effectively limits the sample covariance matrix to within the theoretical covariance matrix by minimizing the error terms while effectively reducing the dependency on snapshot values and noise level variability.

### 3.3. Practical Implementation for DOA Estimation Error Minimization Using Sample Covariance Matrix

Therefore, the diagonal element values of the theoretical covariance matrix can be estimated by the average of the estimated covariance matrix diagonal elements denoted as ${\tilde{R}}_{xx}\left(i,i\right)$ and is defined as

(11) $${\tilde{R}}_{xx}\left(i,i\right)=\frac{\mathrm{tr}\left({\hat{R}}_{xx}\right)}{M}\;,$$

where $\mathrm{tr}\left({\hat{R}}_{xx}\right)$ denotes the trace of the sample covariance matrix, ${\hat{R}}_{xx}$.

Note that the trace of the matrix ${\hat{R}}_{xx}$ is the sum of its complex eigenvalues, and it is invariant to a change of basis. Note that, unlike standard diagonal-loading utilization, where the factor is always generalized and static, our proposed method in (11) is adaptive to its application needs and environmental scenarios such as the SNR, the number of snapshots used, and antenna array geometry.

Using the same observation, the standard deviation of the diagonal elements can also indicate the covariance matrix estimation error. The method of using standard deviation to approximate the estimation error has been used in many past covariance matrix reformulations, such as in [33,34]. In an error-induced scenario, the higher the standard deviation, the higher the variability along the matrix diagonal within that estimated sample, leading to a higher DOA estimation error. Therefore, this assumption can be expressed as

(12) $$\emptyset =\mathrm{SD}\left(\mathrm{diag}\left({\hat{R}}_{xx}\right)\right),$$

where SD(·) means the standard deviation and diag(·) is the diagonal elements of the matrix.

Therefore, we can replace the error term, μ, which is an unknown value, with the standard deviation error identifier, ∅. From (12), an ideal and optimal diagonal loading value to improve DOA estimation via the modified sample covariance matrix should satisfy the following constraint

(13) $$\emptyset \geq \varepsilon_{DL}\ll {\tilde{R}}_{xx}\left(i,i\right),$$

where we can set $\varepsilon_{DL}=\emptyset$ as an initialization value.

Finally, we combine the constraints in (13) onto the sample covariance matrix equation in (6), taking into account the assumption in (7), which is presented as

(14) $${\hat{R}}_{DL}={\hat{R}}_{xx}+\varepsilon_{DL}I.\;\;\;$$

To that end, as the steering vector, $\hat{a}\left(\theta \right)$ is embedded into the received signal matrix, there is a need to extrapolate $\hat{a}\left(\theta \right)$ before applying (14). Therefore, we propose to use a broad initial DOA estimate to obtain $\hat{a}\left(\theta \right)$. This can be done by initiating a rough estimation of the DOA using well-known subspace-based techniques such as root-MUSIC [35]. Then, we can approximate the first steering matrix, $\hat{a}\left(\theta \right)$, as our initial bound estimates. A benefit of extrapolating the steering vector is enabling sufficient system robustness from undesired noise, assuming that the DOA does not deviate and remain static at an instantaneous snapshot that amplifies the steering vector parameter [36]. Furthermore, in a real-world application, the only prior information required to perform good DOA estimation is the knowledge of the antenna array geometry and the angular sector in which the actual steering vector lies [36]. If the incident angle of the signal remains static, then the last (M−L) eigenvalues and their corresponding eigenvectors of the new covariance matrix are invariant. Given the hypothesis, (14) is expanded further with the inclusion of the steering vector estimates, which can be represented as

(15) $${\hat{R}}_{DL}=\left({\hat{R}}_{xx}+\varepsilon_{DL}I\;\right)+a\left(\theta \right)a{\left(\theta \right)}^{H}.$$

Next, we predefine a set tolerance value, δ, where the expected DOA does not deviate between +/−5 degrees. However, this can be scenario-dependent based on the application and the effective beamwidth of the antenna used. For example, a wide beamwidth antenna may have a high tolerance for DOA estimation, whereas a narrow beamwidth-based antenna requires a small tolerance for practical DOA estimation. Then, we set a mathematical constraint between the initial DOA, $\theta_{init}$, and estimated DOA, $\theta_{est}$ with the tolerance value, δ, which can be interpreted as

(16) $$∥\theta_{est}-\theta_{init}∥\;<\;\delta .$$

Algorithm 1 presents a flowchart summary of our proposed algorithm, the CbSS DOA estimation technique. We first obtain the signal, **X**, as in (1) in matrix form. Next, we form the initial sample-based covariance matrix as in (4). We then use root-MUSIC as our initial DOA estimation method for the steering vector to be used in (15). In parallel, we define a predetermined tolerance range that does not overflow the angular expectation of our expected DOA with reference to the initial estimates determined in the previous stage.
**Algorithm 1** Compute DOA using CbSS Algorithm**Require:** Incoming Data Matrix, **X**, *δ* 1: **procedure** CbSS(*θ*) 2: $\;\mathrm{Determine}\;{\hat{R}}_{xx}$ = $\frac{1}{K}XX^{H}$ from **X** 3: $\;\mathrm{Obtain}\;\mathrm{Initial}\;\mathrm{DOA}\;\mathrm{Estimate},\;\theta_{\mathrm{init}}\;\mathrm{from}\;{\hat{R}}_{xx}$ 4: **while**
*θ_est_* is being determined **do** 5:  Estimate $\hat{a}$ (*θ*) from *θ_init_* 6:  $\;\mathrm{Calculate}\;{\tilde{R}}_{xx}$ = *tr*(${\hat{R}}_{xx}$))*/M* 7:  $\;\mathrm{Calculate}\;\varphi \;=\;\mathrm{Std}(\mathrm{diag}({\hat{R}}_{xx}$)) 8:  $\;\mathrm{Set}\;\mathrm{Initial}\;\mathrm{DL}\;\mathrm{value},\;\varepsilon_{DL}$= *φ* 9:  $\;\mathrm{Calculate}\;\mathrm{Modified}\;\mathrm{Covariance}\;\mathrm{Matrix},\;\hat{R}{}_{\mathrm{DL}}=({\hat{R}}_{xx}$ + $\varepsilon_{DL}+\hat{a}(\theta )\hat{a}(\theta ){}^{H}$ 10:  $\;\mathrm{Obtain}\;\mathrm{Estimated}\;\mathrm{DOA}\;\theta_{\mathrm{est}}\mathrm{from}\;{\hat{R}}_{DL}$**.** 11:  **end while** 12: $\;\mathrm{if}\;\theta_{est}-\theta_{init}<\delta$ **then** 13:  end procedure 14: **else** 15:  **loop** 16:   $\;\mathrm{Find}\;\emptyset \geq \varepsilon_{DL}\ll {\tilde{R}}_{xx}$ 17:   Update Steering Vector Estimate, $\hat{a}$(*θ*) 18:   **repeat** 19:    $∥\theta_{est}-\theta_{init}∥$ 20:   $\;\mathrm{until}\;∥\theta_{est}-\theta_{init}∥\;$*< δ* 21:   **end loop** 22:  **end if** 23: **end procedure**

Moreover, the tolerance factor plays a crucial role in the final DOA output because it determines the initial and estimated DOAs. The tolerance factor is vital as it governs the final DOA estimates. For example, the delta has a linear relationship between estimation accuracy and computational time. When delta is low, it leads to higher estimation accuracy but the computational time expense of determining the final DOA. Alternatively, when the delta value is high, it leads to a significantly faster computational time while sacrificing the DOA estimation accuracy. This will be studied further in the simulation section. Depending on the use-case of our proposed algorithm, the end-user can set the appropriate delta values that suit the environment and criticality of the different factors. We conduct the DOA estimation as presented in [21] to determine the estimated steering vector. DOA estimation is then calculated using a modified polynomial root-solving technique that is efficient and with high estimation accuracy. A preliminary analysis of this technique has been demonstrated in [21] for reference. In addition, our CbSS technique allows the flexibility of both multiple and single snapshot scenarios by adapting and manipulating the snapshot variable, K. The following section will present the estimation performance of varying the snapshots and SNR.

## 4. Simulation Results and Discussion

In this section, numerical examples are provided to substantiate the effectiveness of the proposed method. The comparisons are carried out in different performance metrics such as estimation accuracy, computational efficiency, and adaptability to various scenarios. As highlighted before, a compacted size antenna array is needed to ease real-world implementation [30]. Thus, a small-scale ULA with half-wavelength inter-element spacing is considered [30], and the number of antenna array element sensors is M=4 unless otherwise stated. We assume a narrowband signal impinging onto the array from a far-field source. In addition, for simplicity, we assume that the signal source is static in space and does not change with time for all simulation scenarios with only an AWGN interference in the simulation environment within line of sight. The simulation environment is based on a downlink, line-of-sight (LOS) channel model between the receiver and transmitter. The noise data were formed using a normally distributed random number generator in MATLAB that complies with the AWGN model. In our model, the signal matrix, S is assumed to be of a normalized random power while N is modeled as an additive white Gaussian noise (AWGN) interference. In summary, only the received data X is known, whereas the individual parameters A, S, and N are unknown to the DOA estimator because it is randomized in the simulation. Without loss of generality and simplicity, the impinging signal source has a plane-wave characteristic.

The SNR which is used in the simulation is defined as

(17) $$\mathrm{SNR}=\frac{1}{M}\overset{M}{\underset{m=1}{\sum }}\frac{p}{q_{m}},\;$$

where p and $q_{m}$ represent the signal and noise power at the mth array element, respectively.

The SNR equation in (17) corresponds to all sensors’ averaged SNRs and generalizes the definition for uniformed noise levels upon reception at the linear antenna array system. To further examine the performance of our proposed estimator, the standard deviation performance is observed against a range of SNR values and the Cramer-Rao Bound (CRB) [37]. The CRB is a useful statistical comparison tool for the accuracy of parametric methods as it provides a lower bound on the accuracy of any unbiased estimator.

Lastly, all the covariance matrices were simulated using MATLAB 2020b on a Windows 10 PC with a quad-core i7 CPU with 16 GB RAM. A total of 1000 randomized Monte-Carlo simulation trials were used to determine the simulation results. In addition, as it is beyond the scope of this paper, we assume that the number of signals is known a priori. Our proposed CbSS estimator will be evaluated against root-MUSIC [35] and the state-of-the-art partial relaxation (PR) [13] approach in all simulation scenarios for consistency. Table 1 provides a summary of the crucial parameters used in the simulation. In addition, all root mean square error (RMSE) is calculated up to two significant figures per simulation cycle to highlight the high-resolution performance across all demonstrated techniques. The RMSE equation is defined as:

(18) $$\mathrm{Root}\;\mathrm{Mean}\;\mathrm{Square}\;\mathrm{Error}\;\left(\mathrm{RMSE}\right)=\sqrt{\frac{1}{Q}\overset{Q}{\underset{i=1}{\sum }}\left[\frac{{\left(\theta_{i_{1}}-{\hat{\theta }}_{i_{1}}\right)}^{2}+\cdots +{\left(\theta_{i_{L}}-{\hat{\theta }}_{i_{L}}\right)}^{2}}{L}\right]},$$

where L is the number of signal sources as before, Q is the number of simulation data points, $\theta_{i}$ is the actual DOA, and ${\hat{\theta }}_{i}$ is the estimated DOA.

### 4.1. DOA Estimation Accuracy for Single Signal Source and Multiple Finite Snapshots

**Figure 1:**
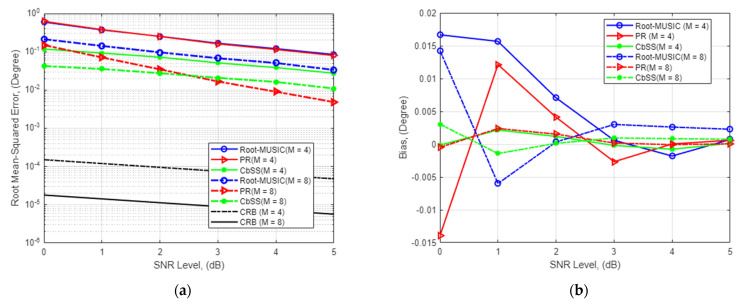

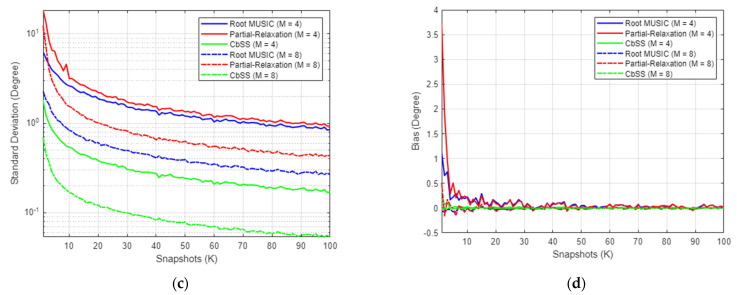
(**a**) SNR-RMSE performance for *M* = 4 and *M* = 8 where the number of snapshots *K* = 100. (**b**) Bias comparison for *M* = 4 and *M* = 8 where the number of snapshots *K* = 100 with varying SNR. (**c**) Standard deviation where *M* = 4 and *M* = 8 against the number of snapshots *K* ranging from 1-100. (**d**) Bias performance comparison where *M* = 4 and *M* = 8 against the number of snapshots *K* ranging from 1 to 100.

Figure 1a presents the RMSE of the DOA estimation against varying SNR ranging from 0 to 5 dB for root-MUSIC, partial relaxation, and our proposed CbSS technique where the number of snapshot samples, K=100, and the number of sensor array elements M=4 and M=8. It is worth highlighting that we chose to compare our proposed technique against root-MUSIC due to the likeliness of algorithm steps with improved estimation performance and the PR approach with its excellent and fast estimation performance.

As depicted in Figure 1a, our proposed CbSS technique exhibits superior performance than root-MUSIC and PR, particularly in lower SNR threshold due to the noise and error-suppressing factor in (15) and the array steering vector’s optimal accuracy defined in (16).

To discuss the finding of the current state-of-the-art performance of the PR approach, at higher SNR (>2 dB), PR outperforms root-MUSIC, albeit slightly insignificant with a small performance margin difference. Focusing on *M* = 4, when SNR = 5 dB, the root-MUSIC, PR, and CbSS presented an absolute RMSE of 0.084°, 0.079°, and 0.028° respectively. This yields a relative estimation performance gain of our proposed CbSS technique of 66.7% and 64.6% compared to root-MUSIC and PR, respectively. At the lowest SNR (0 dB), the three techniques present an RMSE DOA estimation of 0.59°, 0.62°, and 0.12°. These results yield a 79.7% and 80.6% relative estimation performance difference compared to root-MUSIC and PR.

Next, we observe the performance when *M* = 8. When SNR = 0 dB, the root-MUSIC, PR, and CbSS have an RMSE performance of 0.21°, 0.15°, and 0.042°, respectively. This yields a relative estimation performance percentage difference of 80% and 72% when compared between CbSS and root-MUSIC and PR. When SNR = 5 dB, the RMSE difference presents 0.034°, 0.048°, and 0.011° for the three techniques. These results also yield a relative estimation performance difference of 67.6% and 77.1% between CbSS and root-MUSIC and PR.

The supplement Figure 1a,b presents the statistical bias performance of the three DOA estimation techniques with the same simulation parameters to observe the underlying quantitative parameter being investigated. Focusing on *M* = 4, it can be observed that CbSS has a minor variation of bias across the SNR range while approaching minimal bias at a lower SNR of 1.8 dB as compared to root-MUSIC and PR. In a worst-case scenario, when SNR = 0 dB, root-MUSIC, PR, and CbSS are 0.017°, −0.014°, and −0.00014°, respectively. Note that when SNR = 5 dB, the bias approaches negligible levels. Looking at the comparison when *M* = 8, and SNR = 0 dB, root-MUSIC, PR, and CbSS present a bias of 0.014°, −0.0049°, and 0.0031°, respectively. Root-MUSIC presents a significantly lower bias when the number of sensor array elements doubles. The PR approach still has a considerably higher bias when the number of array elements is smaller. In addition, as the PR approach only prioritizes the signal of interest and does not consider the sensor array number and noise environment [22], the PR performance in a low SNR environment is sub-optimal at best but performs significantly better at higher SNR. As the SNR increases, the noise and signal subspace separation is significant and easily differentiated for each method. In addition, as the number of sampling snapshot values increases, the bias starts to be negligible regardless of the methods. Overall, CbSS outperforms the other techniques in terms of statistical bias, proving that the estimation results are stable and predictable.

Figure 1c,d presents the DOA estimation performance among the three techniques in terms of its standard deviation against varying snapshots and its statistical bias performance, respectively. The simulation parameters are the same as before; however, the SNR value remains fixed at 0 dB to observe the DOA estimation performance variation in different snapshot values. Overall, it can be seen that CbSS outperforms root-MUSIC and PR in the case of varying snapshots with similar performance as before. In the scenario depicted in Figure 1c,d, it is evident that the higher the number of snapshots, the lower the standard deviation and statistical bias.

### 4.2. DOA Estimation Accuracy for Multiple Uncorrelated and Coherent Signal Sources

In the second experiment, CbSS performance is observed when multiple signal sources are impinging onto the antenna array to demonstrate high-resolution DOA estimation. This will explain the robustness of signal source separation and estimation accuracy. The presented technique has averaged the RMSE and bias between the two signal sources, and the numerical results are presented. In addition, varying snapshot against standard deviation is also presented against a varying number of antenna elements. In the first subsection, we present an uncorrelated signal source scenario where we observe the difference in performance for varying signal source separation and the number of antenna elements. Likewise, in the second subsection, we present the same simulation scenario as demonstrated in the uncorrelated signal environment but with coherent signal sources.

#### 4.2.1. DOA Estimation Accuracy for Multiple Uncorrelated Signal Sources

**Figure 2:**
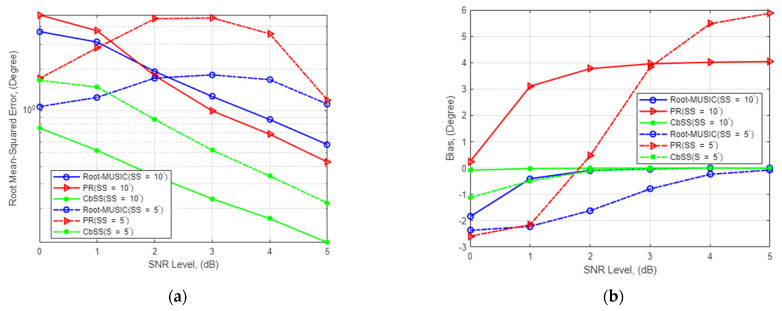

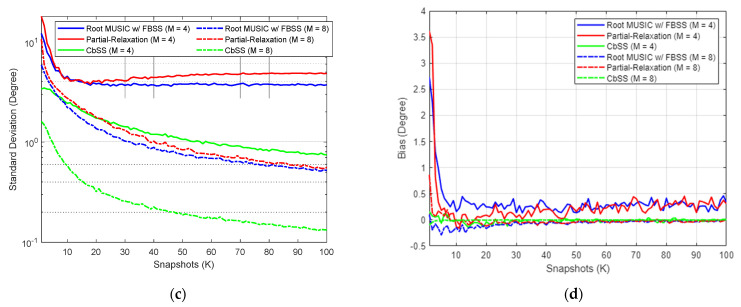
(**a**) SNR-RMSE estimation performance for *M* = 4 with a fixed number of snapshots *K* = 100 for uncorrelated signal source separation of 5 and 10 degrees. (**b**) Bias performance comparison for *M* = 4 with a fixed number of snapshots *K* = 100 for uncorrelated signal source separation of 5 and 10 degrees. (**c**) Standard deviation of DOA estimation comparison against varying snapshots for *M* = 4 and *M* = 8, fixed SNR = 0 dB with uncorrelated signal source separation of 10 degrees. (**d**) Bias comparison against varying snapshots for *M* = 4 and *M* = 8, SNR = 0 dB with uncorrelated signal source separation of 10 degrees.

Figure 2a,b presents the numerical results with multiple degrees of uncorrelated signal source separation and the statistical bias where the array elements *M* = 4, respectively. Our proposed CbSS estimation technique is consistent in performance compared to a single-source environment in a multiple signal source environment. There is an estimation performance decrease of two cubic degrees between a single source and multiple signal source scenarios. It is consistent across all the techniques when the signal source separation is >$10^{{}^{\circ}}$. It is important to note that while the estimation accuracy is high, the algorithm still needs to abide by the M>L constraint.

It is noteworthy that our proposed CbSS technique still performs consistently with 10° signal source separation. However, there is a performance degradation of 25.5% when comparing 5° and 10° signal source separation, respectively. The inconsistent erratic DOA estimation performance is due to the correlation matrix binding with correlated matrix cell inputs. Erratic performance suppression presents the critical advantage of our proposed technique as it can differentiate and solve the two separate signal sources as they approach each other.

Figure 2c,d present the numerical standard deviation against a varying number of snapshots. In this scenario, the SNR remains fixed at 0 dB, and the uncorrelated signal source separation was set at $10^{{}^{\circ}}$. Figure 2c shows that the higher the number of snapshots, the lower the standard deviation. From the results, CbSS presented the lowest standard deviation compared to root-MUSIC and PR, regardless of the number of antenna elements.

#### 4.2.2. DOA Estimation Accuracy for Multiple Coherent Signal Sources

**Figure 3:**
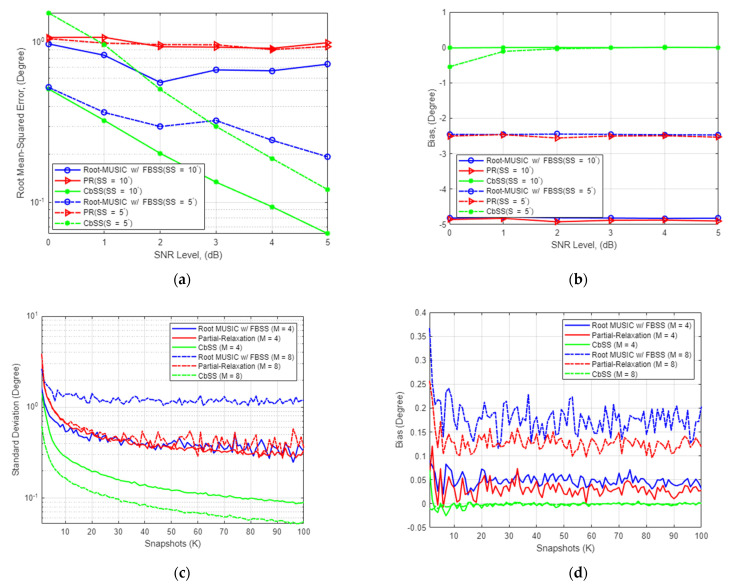
(**a**) SNR-RMSE estimation performance for *M* = 4 with a fixed number of snapshots *K* = 100 for coherent signal source separation of 5 and 10 degrees. (**b**) Bias performance comparison for *M* = 4 with a fixed number of snapshots *K* = 100 for coherent signal source separation of 5 and 10 degrees. (**c**) Standard deviation of DOA estimation comparison against varying snapshots for *M* = 4 and *M* = 8, fixed SNR = 0 dB with coherent signal source separation of 10 degrees. (**d**) Bias comparison against varying snapshots for *M* = 4 and *M* = 8, SNR = 0 dB with coherent signal source separation of 10 degrees.

We observe the scenario where the signal sources of equal power are coherent. In other words, coherent signals of interest have the same phase and frequency and a linear relationship. We reenact the same simulation environment in the coherent signal simulation scenario as presented in Figure 3. In addition, we employ the forward-backward spatial smoothing (FBSS) for the root-MUSIC technique as this technique is well-known for identifying coherent signals relatively well [38]. In addition, the FBSS application onto root-MUSIC does not make a difference in performance when applied to a coherent signal environment.

Figure 3a presents the SNR-RMSE DOA estimation performance for *M* = 4 with a fixed number of snapshots *K* = 100 for coherent signal source separation of 5 and 10 degrees. CbSS has the lowest RMSE compared to root-MUSIC and PR, with similar estimation performance compared to an uncorrelated scenario. However, all techniques have an estimation performance degradation of approximately 10% while sustaining a higher RMSE at higher SNR when compared to an uncorrelated signal scenario. This is expected due to the difficulty of accurately isolating and decomposing the signal and noise subspace. Figure 3b presents the statistical bias with the same simulation parameters as presented in Figure 3a. Our proposed CbSS technique has an almost negligible bias DOA estimation performance compared to root-MUSIC with FBSS and PR techniques. The bias results support past literature that the root-MUSIC and PR technique, although accurate in terms of RMSE, are susceptible to a high level of bias.

Figure 3c presents the standard deviation of DOA estimation comparison against varying snapshots for *M* = 4 and *M* = 8, with a fixed SNR value of 0 dB and a coherent signal source separation of 10 degrees. Similar to Figure 2c, it is evident that the higher the number of snapshots, the more accurate the DOA estimation is. Nevertheless, our proposed CbSS technique has the lowest standard deviation compared to root-MUSIC and PR regardless of the number of elements. Figure 3d presents the statistical bias comparison for the same simulation environment as demonstrated in Figure 3c. Clearly, due to the coherent signal environment, there is a lot of bias jitter across the three techniques. Based on the results, CbSS presents almost negligible bias again than root-MUSIC and PR. However, compared to an uncorrelated scenario, as shown in Figure 2d, it converges toward 0 at a much higher SNR level. At the same time, the root-MUSIC and PR techniques maintain undesirably high bias values.

In summary, the PR method performs the worst in a coherent signal environment. This is because for the PR approach, instead of enforcing the entire structure on the steering vector when formulating the DOA estimation problem, only the structure of one source of interest is preserved while other additional sources are relaxed. In a situation where there are multiple sources, PR can only be effective when the sources are uncorrelated [13]. This is evident from Figure 2d, where PR performs the worst compared to CbSS and root-MUSIC across the wide range of SNR.

Second, the root-MUSIC with FBSS performs relatively well in terms of bias performance due to the spatial averaging. The difference between the estimated and actual DOA is much smaller in a coherent signal environment when FBSS is employed for root-MUSIC. Based on the results illustrated in Figure 2c, our proposed CbSS technique outperforms the spatially smoothed root-MUSIC approach by presenting a significantly lower bias, especially at high SNR. When the coherent signal source separation is at its worst of 5 degrees where SNR is −20 dB, our proposed CbSS technique has a bias of −2.2 degrees. Our proposed method approaches near 0 bias when SNR is >0 dB in both signal source separation environments. The ability to resolve coherent signal here is possible due to the highly recursive updates and diagonal loading factor applied to the steering vector as shown in (15) and (16).

### 4.3. Estimation Accuracy for Single Signal Source and Single Snapshot

**Figure 4:**
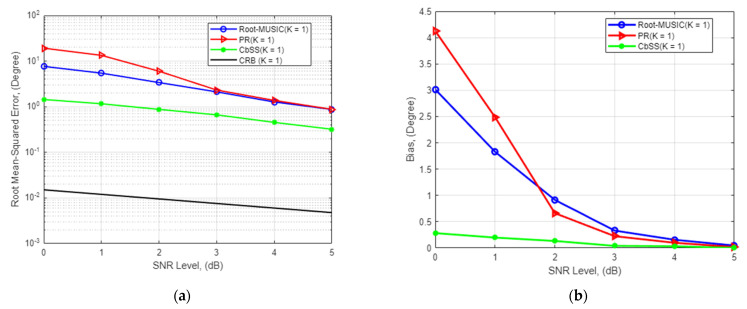
(**a**) SNR-RMSE for single snapshot comparison where the number of antenna array elements *M* = 4 and *K* = 1. (**b**) Statistical bias performance comparison across the demonstrated techniques for single snapshot scenario.

The proposed technique estimation performance is observed in the third experiment under a single snapshot scenario where M=4 and K=1 as worst-case scenarios. All other parameters are the same and can be referred to in Table 1. Like the performance of a multiple finite snapshot sample scenario, our proposed CbSS technique outperforms the root-MUSIC and PR approach. With reference to Figure 4a, when SNR = 0 dB, the RMSE difference relative to the CRB in a single snapshot scenario is 7.7°, 19°, and 1.4° for the root-MUSIC, PR approach, and our proposed CbSS techniques, respectively. This yielded a performance percentage difference of 81.9% and 92.6% between CbSS and compared against root-MUSIC and PR. When the SNR = 5 dB, the RMSE difference is 0.87°, 0.88°, and 0.32°  for the three estimation techniques, respectively. This yields a performance percentage difference of 63.2% and 63.6%, respectively. The simulation results prove that the CbSS technique is robust even in a single snapshot scenario providing satisfactory DOA estimation accuracy with the help of the accurate array steering vector estimation and the noise and estimation error suppressing factor in (10).

Figure 4b presents the statistical and focused biased plots for our proposed CbSS technique for K=1 and M=4. CbSS trumps the bias difference comparison and performance in a single snapshot scenario compared to the root-MUSIC and PR approach with an absolute maximum bias estimation of 0.0018° at the lowest SNR of 0 dB. We want to highlight that our proposed CbSS technique approaches an unbiased-like performance at a lower SNR of 3.5 dB under a single snapshot environment. An essential factor to note about the PR approach is that although it has a very high bias at low SNR compared to the other techniques overall, the computational time of the PR approach is deficient, which will be addressed in the next section. In addition, since the PR approach does not fully consider the entire signal and noise subspaces at low SNR, this technique does not perform as well as CbSS and the root-MUSIC method to prioritize computational complexity and calculation time of the final DOA estimates.

### 4.4. Parametric Performance Impact

Table 2 presents the performance comparison between peak DOA estimation accuracy and computational time for the CbSS technique. The simulation is based on a single source and single snapshot scenario with the same parameters in Table 1 based on the delta value in (16). As demonstrated in Table 2, the higher the delta value, the faster the DOA estimation sequence is completed, but this is at the expense of peak accuracy. Likewise, a lower delta value results in higher peak estimation accuracy but at the cost of computational time to completion. Depending on the application environment and priority, the end-user of CbSS has the flexibility to select the appropriate delta. For example, in a transportation application where high-speed targets are of concern, the user may choose delta values of 0.5 where there is a need for quick DOA estimation. Alternatively, in a scenario where there are slow speed targets, such as in congested traffic, the user may select delta values of 0.1 for higher DOA estimation accuracy.

## 5. Conclusions

This paper investigates the problem with DOA estimators in low SNR scenarios with uniformed linear arrays in the presence of noise and the practicality of using a single snapshot to reduce computational complexity and time. Our proposed algorithm’s simulation results are consistent and perform well in low SNR scenarios by utilizing a well-approximated steering vector to modify the input covariance matrix. Our proposed method is robust in estimation stability and can offer satisfactory DOA estimation performance. The simulation results have demonstrated that our proposed CbSS technique performs best among the three presented methods (root-MUSIC, PR, CbSS). The simulation work has been shown for multiple and single snapshot scenarios with adequate overall computational time compared to an existing state-of-the-art method like the partial relaxation approach. In addition, our proposed CbSS technique presented good estimation performance in a multi-signal scenario even with signal separation of 5°. The essential advantage of our proposed CbSS technique is efficient covariance matrix data collection coupled with accurate DOA estimation. Our results present improved DOA estimation accuracy at lower SNR than the geometric-based DOA estimators with lower statistical bias. However, at higher SNR, the geometric-based approach still presents an improved signal resolution for multiple sources compared to our subspace-based technique.

Nevertheless, our proposed method is applicable in scenarios where SNR is low and needs a small-scale and lightweight antenna array localization application and systems. One industry that urgently requires accurate DOA estimation is the intelligent transportation system (ITS) network. Our proposed CbSS technique enables fast and precise network connectivity from stationary base stations and dynamically moving vehicular systems as required in ITS applications. As part of future work, practical experiments will be conducted to validate our proposed CbSS technique in line with past DOA-based experiment validation studies, as demonstrated in [39,40]. Based on [39,40], the validation of the proposed CbSS technique can be carried out using field programmable gate array (FPGA) prototyping platforms. In past DOA estimation literature, such as [39,41], practical experiments have demonstrated that it can meet real-world applications with sufficient computational complexities of the same subspace-based class of DOA estimators. In addition, a comprehensive comparison and performance analysis against geometric-based DOA estimators can be carried out to compare the estimation robustness and accuracy.

## Figures and Tables

**Table 1:** Common simulation parameters.

Carrier Frequency, $f_{c}$	5500 MHz
Antenna Geometry	Uniformed Linear Array
Array Inter-Element Spacing	λ/2, where λ is the wavelength of $f_{c}$ in meters
No. of Array Elements, M	4, 8
Simulation Sample	1000
Angle of Interest	35 Degrees (Single Signal Source) 35 ± 10 Degrees (Double Signal Source)
SNR Range	−20 dB to 10 dB
Tolerance, δ	+/−0.01

**Table 2:** Comparison of varying delta values.

Delta, *δ*	0.01	0.1	1	10
Peak Accuracy	98.7%	91.2%	79.2%	60.5%
Computational Time/cycle (millisecond)	0.23	0.19	0.12	0.08

## Data Availability

Not applicable.
